# Platelet count and indoor cold exposure among elderly people: A cross-sectional analysis of the HEIJO-KYO study

**DOI:** 10.1016/j.je.2016.12.018

**Published:** 2017-06-20

**Authors:** Keigo Saeki, Kenji Obayashi, Norio Kurumatani

**Affiliations:** Department of Community Health and Epidemiology, Nara Medical University School of Medicine, Nara, Japan

**Keywords:** Platelet count, Indoor temperature, Elderly

## Abstract

**Background:**

Excess mortality from cardiovascular disease during cold seasons is a worldwide issue. Although some physiologic studies suggests that platelet activation via cold exposure may cause an increased incidence of cardiovascular disease in winter, the influence of indoor cold exposure in real-life situations on platelet (PLT) count remains unclear.

**Methods:**

A cross-sectional study was conducted among 1095 elderly individuals. After obtaining a venous sample in the morning, indoor temperature of participants' home was measured every 10 min for 48 h. The mean indoor temperature while the participants stayed at home was calculated. All measurement was conducted during cold seasons (October to April) from 2010 to 2014.

**Results:**

The mean age of the 1095 participants was 71.9 years. They spent 87.3% of the day at home (20 h 27 min). A 1 °C lower daytime indoor temperature was associated with a significant increase in PLT count of 1.47 × 10^9^/L (95% confidence interval, 0.39–2.56 × 10^9^/L). Compared with the warmest tertile group (20.1 [standard deviation {SD}, 0.09] °C), the coldest group (11.7 [SD, 0.12] °C) showed a 5.2% higher PLT count (238.84 [SD, 3.30] vs. 226.48 [SD, 3.32] × 10^9^/L; P = 0.01), even after adjusting for basic characteristics (age, gender, body weight, and smoking), antihypertensive medication, comorbidities (diabetes, estimated glomerular filtration rate), socioeconomic status (household income and education), day length, and outdoor temperature.

**Conclusions:**

We found a significant and independent association between lower indoor temperature and higher PLT count among elderly in winter.

## Highlights

• Lower daytime indoor temperature was associated with higher PLT count in winter.
• The association was independent of outdoor temperature and socioeconomic status.
• The 1095 elderly participants spent the majority of their time at home (87.3%).

## Introduction

Excess mortality during cold seasons is an issue worldwide.^1^, ^2^, ^3^, ^4^, ^5^ Higher mortality in winter is predominantly attributable to cardiovascular disease (CVD) and respiratory infections.^6^ Cold exposure-induced activation of the hemostatic system has been suggested as a potential cause of increased CVD incidence in winter.^7^

Platelet (PLT) count is an important determinant of PLT aggregation. *In vitro*, whole blood aggregation is associated with PLT count not only among healthy participants^8^, ^9^ but also among patients with ischemic heart disease.^10^ In addition, antiplatelet therapy significantly decreased events of thrombotic diseases, such as acute myocardial infarction and stroke.^11^ According to epidemiological studies, increased PLT count is associated with an increased incidence of coronary heart disease^12^, ^13^ and all-cause mortality.^14^

An increased PLT count induced via cold exposure for 6 h was reported in an experimental study among eight healthy young participants.^7^ Consistent with this experimental study, observational study among 1001 men aged 50–65 years showed a significant association between a 16.0 × 10^9^/L-higher PLT count and a 16 °C lower outdoor temperature at the day of the test.^15^

However, the quantification of cold exposure using outdoor temperatures is not sufficient among elderly individuals, a high-risk group for excess mortality during winter, because they spend the majority of their time (>85%) at home.^16^ Indeed, ambulatory blood pressures among elderly individuals were more strongly associated with indoor temperature than outdoor temperature.^16^

An inter-regional study reported that excess mortality during winter was observed to be higher in southern European countries with mild winter climates than in northern countries with severe cold climates.^5^ As a potential explanation of these results, an ecological study showed higher indoor temperature among northern cities in Europe than southern cities.^17^ Furthermore, the changes in health outcomes associated with indoor temperature suggest a preventable fraction by modification of the indoor environment. As far as we know, there is no previous study about the association between indoor temperature and PLT count.

The purpose of the present study was to investigate the association between indoor cold exposure and PLT count among elderly individuals.

## Methods

This cross-sectional analysis was performed on the baseline data of a community-based cohort study: the Housing Environments and Health Investigation among Japanese Older People in Nara, Kansai Region (HEIJO-KYO) study.^18^ We recruited 1127 volunteers who met the inclusion criteria of the present study: males or females, aged 60 years or greater, and home dwelling from 2010 to 2014. Of these, we conducted measurements during winter (October to April) from 2010 to 2014 in 1122 participants. After excluding participants with missing PLT data (n = 11) or missing indoor home temperatures (n = 16), 1095 participants remained for the present study. Prior to participation in the HEIJO-KYO study, all participants provided written informed consent. The study protocol was approved by the Nara Medical University Medical Ethics Committee.

An overnight fasting venous sample was collected with minimum stasis (approximately 1–3 min) in the morning. To prevent clotting, blood samples were immediately transferred to plastic tubes containing ethylene-diamine-tetra-acetic acid (EDTA) and stored in a cold box until measurement. PLT counts were measured at a commercial laboratory (SRL Co. Inc., Tokyo, Japan).

After obtaining venous samples, indoor temperature measurements for 48 h were initiated at noon. Indoor temperatures in the living room and bedrooms of participants' home were measured at approximately 60 cm above the floor. We defined the living room as where the participants spend the most of their awake time. Bed temperatures were measured at the center of the bed, 50 cm from headboard, beneath the top sheet. For participants who sleep not in bed but on a Japanese-style “futon” mattress, we set the thermometer at 50 cm from the head-side edge of the mattress beneath the top sheet. Indoor and bed temperature were measured every 10 min using the Thermochron iButton DS1922L (Maxim Integrated, San Jose, CA, USA) with a measurement accuracy of ±0.5 °C between −10 °C and +65 °C at a 0.0624 °C resolution. Outdoor temperatures of the measurement day at 10-min intervals were provided by the meteorological office in Nara (34 °N). All participants kept a standardized diary logging the time spent in the bedroom and the time spent out of their home.

We regarded living room temperature as the indoor temperature while the participants stay at home, except when participants were in their bedrooms. While the participants stayed in their bedrooms, the bedroom temperature was regarded as the indoor temperature. After identification of the indoor temperature at every 10-min increment based on the self-administered diary, we calculated the mean indoor temperature while the participants stay at home during the daytime (rising time to bedtime), nighttime (bedtime to rising), morning (2 h after rising), and evening (2 h before bedtime). For calculation of indoor temperature, the data on times the participants spent out of home were excluded. As for outdoor temperature, we calculated mean values during the daytime (rising time to bedtime), nighttime (bedtime to rising), morning (2 h after rising), and evening (2 h before bedtime).

Current smoking status, alcohol intake, antihypertensive medication, and socioeconomic status were evaluated using a standardized questionnaire. Missing information was obtained through an interview. Diabetes was defined as fasting plasma glucose ≥126 mg/dL and hemoglobin A1c ≥ 6.5% (according to the National Glycohemoglobin Standardization Program) or current use of antidiabetic medication.

Continuous variables with normal distributions are presented as mean (standard error [SE]). The univariate association between temperatures and PLT count was assessed using a linear regression model. We categorized all participants into three groups (cold, intermediate, and warm groups) by tertile values of daytime indoor temperature (Table 1). Mean PLT count adjusted for potential confounders and 95% confidence intervals (CI) in tertile groups of daytime indoor temperatures were estimated using analysis of covariance (ANCOVA). We assessed multicollinearity using variance inflation factor (VIF). All statistical analyses were conducted using SPSS version 21.0 for Windows (IBM SPSS Inc., Chicago, IL, USA).

**Table 1:** Basic characteristics of 1095 participants by daytime indoor temperature.

	Daytime indoor temperature			*P* for trend
	Cold ≤14.4 °C	Intermediate 14.4–17.9 °C	Warm >17.9 °C	
Number	365	365	365	
Indoor temperature, °C, mean (SE)				
Daytime	11.7 (0.12)	16.2 (0.06)	20.1 (0.09)	<0.01
Nighttime	9.3 (0.16)	12.0 (0.18)	15.7 (0.21)	<0.01
Outdoor temperature				
Daytime	5.7 (0.16)	8.5 (0.22)	11.5 (0.28)	<0.01
Nighttime	2.7 (0.18)	4.3 (0.23)	7.6 (0.27)	<0.01
Bed temperature				
Nighttime	30.1 (0.25)	29.6 (0.27)	30.2 (0.22)	0.15
Day length, min	634.3 (2.10)	651.4 (2.68)	662.8 (2.84)	<0.01
Time spent out of home (>183 min^a^), N (%)	202 (55.3)	168 (46.0)	174 (47.7)	0.04
Basic characteristics				
Age, mean (SE)	72.0 (0.38)	72.0 (0.36)	71.8 (0.37)	0.67
Male, N (%)	157 (43.0)	179 (49.0)	180 (49.3)	0.08
Weight, mean (SE)	56.1 (0.56)	57.4 (0.56)	58.1 (0.53)	0.01
eGFR, mean (SE)	73.3 (0.84)	73.4 (0.78)	70.8 (0.77)	0.03
Current smoker, N (%)	18 (4.9)	16 (4.4)	21 (5.8)	0.61
Ethanol intake (>30 g/day), N (%)	49 (13.4)	61 (16.7)	50 (13.7)	0.92
Antihypertensive medication, N (%)	165 (45.2)	166 (45.5)	159 (43.6)	0.66
Diabetes, N (%)	37 (10.2)	45 (12.4)	45 (12.5)	0.33
House hold Income (≥4 million JPY/year), N (%)	133 (36.4)	144 (39.5)	161 (44.1)	0.04
Education, (≥13 years), N (%)	91 (24.9)	93 (25.5)	104 (28.5)	0.26
Hematologic parameters, mean (SE)				
WBC, 10^9^/L	5.34 (0.07)	5.34 (0.07)	5.40 (0.08)	0.54
RBC, 10^12^/L	4.50 (0.02)	4.51 (0.02)	4.46 (0.02)	0.22
Ht, %	42.7 (0.22)	42.7 (0.21)	42.2 (0.20)	0.08

eGFR, estimated glomerular filtration rate; Ht, hematocrit; RBC, red blood cell; SE, standard error; WBC, white blood cell.

a Median time spent out of home.

To assess the validity of self-reported diary, we compared the time to bed based on the self-reported diary and the objectively measured time when the bed temperature rose. We regarded the time of bed temperature-rise as the first rise of bed temperature over 1.0 °C in 10 min around 6 h of self-reported time to bed (from 3 h before self-reported bedtime to 3 h after self-reported bedtime).

## Results

The mean age of all 1095 participants was 71.9 (SE, 0.22) years, and 516 participants (47.1%) were male. The participants spent the majority of their time at home (median, 87.3% [20 h 27 min]). The validity of the self-administered diary was considered to be acceptable, given the strong correlation between self-reported bedtime and objectively measured the time of bed temperature-rise (r = 0.83).

Mean daytime indoor temperatures in the tertile groups were 11.7 (SE, 0.12) °C, 16.2 (SE, 0.06) °C, and 20.1 (SE, 0.09) °C, respectively (Table 1). Daytime indoor temperature was significantly associated with outdoor temperature, day length, time spent out of home, body weight, estimated glomerular filtration rate (eGFR), and house hold income (Table 1).

**Fig. 1:**
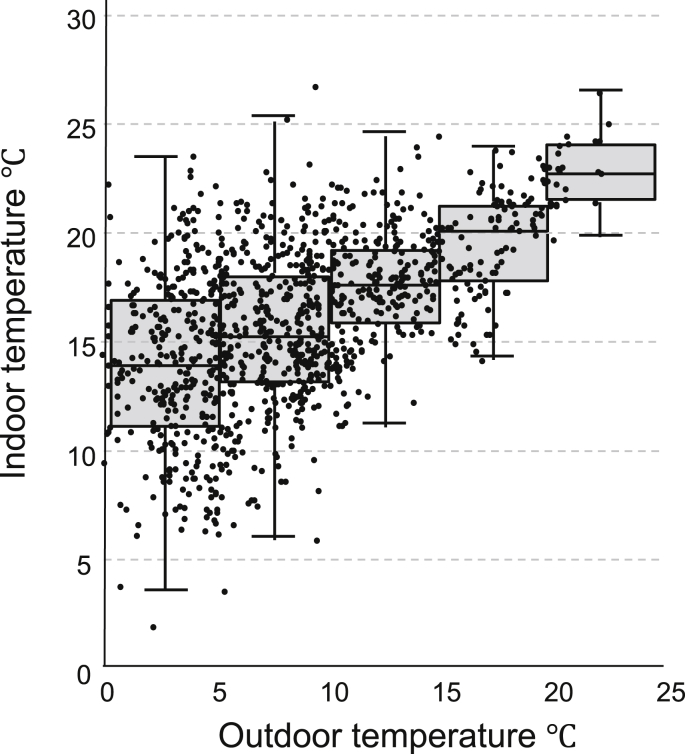
Box and scatter plot of indoor temperature and outdoor temperature in daytime. Box plot shows median and inter quartile range of indoor temperature by outdoor temperature group (<5 °C, 5–10 °C, 10–15 °C, 15–20 °C, and ≥20 °C).

Box and scatter plot of indoor and outdoor temperatures showed higher variability of indoor temperature in cold outdoor temperature than those in warm outdoor temperature (Fig. 1). Medians of indoor temperatures in each range of outdoor temperatures (<5 °C, 5–10 °C, 10–15 °C, 15–20 °C, and ≥20 °C) were 14.0 (interquartile range [IQR], 5.83), 15.2 (IQR, 4.84), 17.7 (IQR, 3.35), 20.2 (IQR, 3.46), and 22.8 (IQR, 2.53), respectively (Fig. 1).

**Fig. 2:**
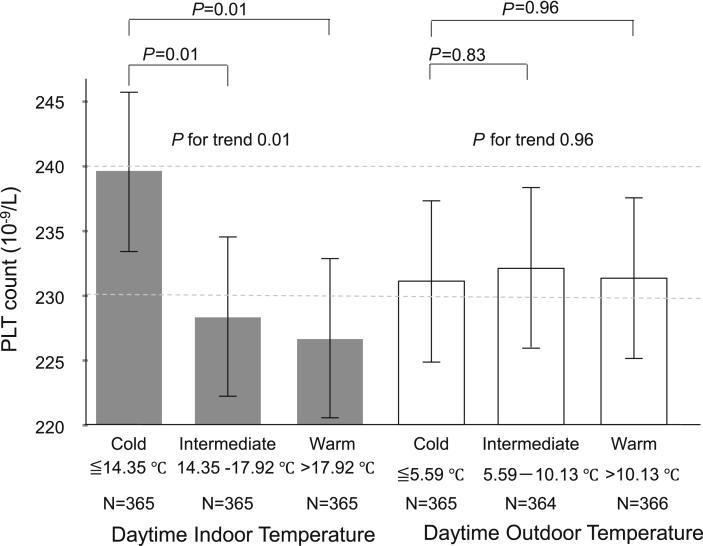
Mean PLT count by indoor temperature and outdoor temperature. Gray column shows mean PLT count by tertile group of indoor temperature, and white column shows mean PLT count by tertile group of outdoor temperature. Error bars shows 95% confidence interval. PLT, platelet.

The mean PLT count of three groups by tertile values of daytime indoor temperature were 239.3 × 10^9^/L (95% CI, 233.2–245.4), 228.2 × 10^9^/L (222.1–234.2), and 226.5 × 10^9^/L (220.4–232.6), respectively (Fig. 2).

Compared with the cold group, PLT count in the intermediate and warm group were significantly lower by 11.1 × 10^9^/L (*P* = 0.01) and 12.8 × 10^9^/L (*P* = 0.01), respectively, with a significant linear trend (*P* for trend = 0.01). In contrast to the indoor temperature, outdoor temperature did not show significant association with PLT count (Fig. 2).

Simple linear regression analysis demonstrated a 1 °C higher daytime indoor temperatures was significantly associated with a lower PLT count by 1.42 × 10^9^/L (95% CI, 0.50–2.33; *P* < 0.01). Morning and evening indoor temperature were also significantly associated with PLT counts. However, daytime outdoor temperature, nighttime indoor temperature, nighttime bed temperature, and day length did not show significant association with PLT count (Table 2).

**Table 2:** Simple linear regression analysis between temperatures and PLT count.

Variables	Regression coefficient (95% CI)^a^	*P* value
Indoor temperatures, °C		
Daytime	−1.42 (−2.33, −0.50)	<0.01
Morning (2 h after rising)	−1.15 (−1.95, −0.36)	<0.01
Evening (2 h before bedtime)	−0.90 (−1.76, −0.03)	0.04
Nighttime	−0.25 (−1.06, 0.55)	0.54
Outdoor temperature, °C		
Daytime	−0.22 (−0.94, 0.49)	0.54
Nighttime	0.05 (−0.67, 0.79)	0.87
Bed temperature, °C		
Nighttime	−0.15 (−0.90, 0.59)	0.69
Day length	−0.01 (−0.08, 0.06),	0.78

CI, 95% confidence interval; daytime, rising to bedtime; Nighttime, bedtime to rising.

a Regression coefficients represent a change of PLT count (10^9^/L) associated with a 1 °C increase of daytime indoor temperature during at home.

After adjusting for age, gender, and hematocrit, a 1 °C higher daytime indoor temperature was significantly associated with a lower PLT count by 1.36 × 10^9^/L (95% CI, 0.45–2.26) (model 1 in Table 3). Model 2, with further adjustment for daytime outdoor temperature, time spent out of home, and day length, also showed a significant association between a 1 °C higher daytime indoor temperature and a lower PLT counts by 1.66 × 10^9^/L (95% CI, 0.58–2.73) (model 2 in Table 3).

**Table 3:** Adjusted mean of PLT counts by daytime indoor temperature.

	Daytime indoor temperature^d^			Adjusted β^e^
	Cold	Intermediate	Warm	
Model 1^a^				
PLT count, 10^9^/L, mean (SE)	238.71 (3.06)	228.57 (3.05)	226.65 (3.06)	−1.36 (−2.26, −0.45)
Difference, 10^9^/L (%)	ref	−10.14 (−4.2%)	−12.06 (−5.1%)	
*P* for difference		*P* = 0.02	*P* = 0.01	
Model 2^b^				
PLT count, 10^9^/L, mean (SE)	239.57 (3.29)	228.67 (3.06)	225.70 (3.29)	−1.66 (−2.73, −0.58)
Difference, 10^9^/L (%)	ref	−10.90 (−4.5%)	−13.88 (−5.8%)	
*P* for difference		*P* = 0.02	*P* = 0.01	
Model 3^c^				
PLT count, 10^9^/L, mean (SE)	238.86 (3.30)	228.84 (3.06)	226.48 (3.32)	−1.47 (−2.56, −0.39)
Difference, 10^9^/L (difference%)	ref	−10.02 (−4.2%)	−12.39 (−5.2%)	
*P* for difference		*P* = 0.03	*P* = 0.01	

CI, confidence interval; SE, standard error.

a Model 1: adjusted for age (per 5 years), gender, and hematocrit.

b Model 2: adjusted for variables in model 1, daytime outdoor temperature, time spent out of home, and day length.

c Model 3: adjusted for variables in model 2, current smoking, ethanol intake (≥30 g/day), eGFR, body weight, antihypertensive medication, diabetes, house hold income, and education.

d The ranges of tertile groups of indoor temperature in daytime: ≤14.4 °C, intermediate: 14.4–17.9 °C, and warm: >17.9 °C.

e Change of PLT count by a 1 °C increase of daytime indoor temperature.

In the fully adjusted model (model 3), with further adjustment for current smoking, ethanol intake (≥30 g/day), eGFR, body weight, antihypertensive medication, diabetes, house hold income, and education, a 1 °C higher daytime indoor temperature was associated with a lower PLT count by 1.47 × 10^9^/L (95% CI, 0.39–2.56). Compared with the cold group, the intermediate group and the warm group showed a significantly lower PLT count by 4.2% (10.02 × 10^9^/L, *P* = 0.03), and 5.2% (12.39 × 10^9^/L, *P* = 0.01), respectively (model 3 in Table 3). After further adjustment for the month of measurement day (January–February vs. others), we found a significant association between PLT counts and indoor temperature independent of seasonal effect (adjusted β = −1.51, *P* = 0.006). In multivariable models, we did not find any variables with variance inflation factor greater than or equal to 10.

## Discussion

To our knowledge, this is the first epidemiologic study investigating the association between indoor cold exposure and PLT count in a real-life setting. We observed a significant inverse association between indoor cold exposure and PLT count. The association remained significant after adjustment for potential confounders, including age, gender, hematocrit, outdoor temperature, day length, time spent out of home, smoking, ethanol intake, body weight, eGFR, antihypertensive medication, diabetes, and socioeconomic status (household income and education). According to the categorical analysis by tertile groups based on daytime indoor temperatures, an average of 8.4 °C lower daytime indoor temperature was associated with a 5.2% higher PLT count. Compared with the crude results (Fig. 2) and the fully adjusted model (model 3, Table 3), we not only found a consistently significant association of PLT count and indoor temperature, but also a similar association of PLT count with 1 °C change of indoor temperature. This may suggest that the effect of confounding factors is small.

The findings of the present study among home-dwelling elderly expand the generalizability of evidence from a previous experimental study. Keatinge et al proposed cold-induced higher PLT counts as a potential cause of excess winter CVD mortality based on the results that a cooling intervention for 6 h in a net bed by rapid air movement at 10 m/s caused an 8% increase in PLT count from 291 × 10^9^/L to 314 × 10^9^/L.^7^ However, the generalizability of the results of this study was limited because of the small sample size (8 healthy young participants), and because the cooling intervention was not usual in real-life situations. Increased PLT count induced by cold intervention in the previous study (8%) corresponds to a higher PLT count by 18.5 × 10^9^/L (231.31 × 10^9^/L [mean PLT count among all participants] × 0.08), and it is associated with a lower daytime indoor temperature by 12.6 °C based on the fully adjusted model of the present study (model 3 in Table 3). In cold outdoor temperature (<10 °C), we observed an inter-personal difference of daytime indoor temperature over 12.6 °C in a real-life situation (Fig. 1).

A strength of the present study is an accurate estimation of cold exposure based on objectively measured indoor temperature. A previous epidemiological study using outdoor temperature as a cold exposure conducted through winter to summer showed that a 16 °C-lower outdoor temperature is associated with a 16.0 × 10^9^/L higher PLT count among 1001 middle-aged men (50–65 years).^15^ However, the study had limitation in assessment of cold exposure, because the influence of outdoor temperature is modified due to heating and housing insulation, especially during the participants' stay indoors. In the present study, we overcame this limitation by accurately estimating cold exposure using indoor temperature among elderly who spent most of the time at home (median, 87.3% [20 h 27 min]). In fact, lower correlation between indoor and outdoor temperatures, particularly in cold climates, shows the difficulty in estimating indoor cold exposure based on outdoor temperature because of inter-personal variability of housing environment (Fig. 1). In addition, our results suggest the modifiable fraction of PLT counts associated with controlling the indoor thermal environment. The second strength of the present study was the relatively large sample size, which allowed the inclusion of a comprehensive list of potential confounders, including patient characteristics, comorbidities, and socioeconomic status.

The finding of a significant association between PLT count and indoor temperatures but not outdoor temperatures was consistent with a previously reported association between ambulatory BP and indoor temperatures but not outdoor temperatures.^16^ The absence of a significant association between outdoor temperature and PLT count in the present study is reasonable because we conducted all measurements only in the cold season and the range of outdoor temperatures was narrow compared with a study conducted though winter to summer.

We found a significant association between PLT count and daytime indoor temperature, but not with bed temperature in nighttime. The reason may be explained by the difference of characteristics of the temperatures. In contrast to the indoor temperature, bed temperature tends to be influenced by body temperature because it is measured at a point closer to the body and in a space that is highly insulated by bedclothes. In fact, we found similar finding form a randomized controlled study comparing the ambulatory blood pressure in normal indoor temperature (24 °C) and colder indoor temperature (14 °C). In the cold exposure group in daytime, a 10 °C-lower indoor temperature caused significantly lower skin temperature and higher ambulatory BP, despite participants' wearing 1.75 kg more clothing (*P* < 0.01) in the morning and 1.45 kg more clothing (*P* < 0.01) in the evening than the control group. In contrast, the influence of indoor cold exposure in nighttime was attenuated due to the insulation of bed clothing.^19^

Although the mechanisms underlying PLT activation in response to cold exposure have yet to be fully elucidated, the short-term and long-term effects of plasma catecholamine induced by sympathetic nerve activity may contribute to this phenomenon.^20^ Cold exposure has been shown to increase plasma catecholamine concentration.^21^, ^22^, ^23^, ^24^, ^25^ As a result of splenic constriction induced by plasma catecholamine,^26^ pooled thrombocytes in the spleen are released into the blood stream, leading to increased PLT count.^27^, ^28^ Although the plasma catecholamine has a short half-life and disappears quickly (within 30 min), a part of the plasma catecholamine is taken up by the PLTs^29^, ^30^ and demonstrates a relatively long-term effect. After an 80-min infusion of epinephrine at 2.75 μg/min, plasma epinephrine concentration was found to return to baseline levels within 30 min; however, PLT epinephrine levels remained elevated above baseline after 200 min.^31^ *In vitro* studies have demonstrated that PLT catecholamine levels play an important role in aggregation.^29^, ^32^ This mechanism is similar to PLT activation in response to upright posture in the morning via catecholamine secretion, which may be associated with a morning surge in CVD incidence.^33^, ^34^ During cold periods, higher PLT count may promote blood coagulation in conjunction with other hemostatic factors known to have serum concentration peaks in winter, such as fibrinogen,^15^, ^35^ tissue plasminogen activator, and von Willebrand factor.^36^

The clinical importance of higher PLT count has been suggested in a prospective cohort study of 487 men with 13.5 years' follow-up. Thaulow et al reported that mortality from coronary heart disease among the highest quartile group of PLT count (median, 298 × 10^9^/L) was 126% higher than other groups (median, 211 × 10^9^/L), independent of age, smoking, cholesterol level, and systolic BP.^12^ Based on this data, the cold group of the present study may be associated with an 17.9% increased risk of coronary heart disease compared with the warm group. Another large-scale cohort study found a significant association between PLT count and all-cause mortality.^14^

The present study had several limitations. First, only PLT count was assessed as a hemostatic factor. Second, we cannot determine the direction of causality between indoor temperature and PLT count from the cross-sectional analysis. As an index of indoor cold exposure at measurement of PLT count, we used the mean value of indoor temperatures in the following day from rising time to bedtime, which may be acceptable based on high repeatability of indoor temperatures on two consecutive daytimes (noon to noon) among 923 participants (correlation coefficient = 0.92). However abrupt change of outdoor temperature may distort the results. Third, we drew venous samples from all participants with stasis. A previous study showed that PLT count collected with 1-min and 3-min stasis showed acceptable correlation with PLT count collected without stasis (r > 0.95).^37^ Fourth, we did not quantify the amount of clothing worn. In a previous randomized controlled trial, we found a significant increase in the amount of clothing worn due to a 10 °C-lower indoor temperature. The influence of indoor temperature on PLT count may be underestimated if the participants in cold indoor temperature wear more clothing than in the present study. Finally, participants were not randomly selected but were volunteers. This may limit the generalization of the findings of the present study to the elderly population as a whole. However, patient characteristics, such as body mass index and eGFR, were similar to those reported in a nationwide survey of randomly selected participants.^38^

In conclusion, we demonstrate a significant inverse association between PLT count and daytime indoor temperature in the homes of elderly individuals during winter.
